# Identification of novel mutations in X-linked retinitis pigmentosa families and implications for diagnostic testing

**Published:** 2008-06-06

**Authors:** John Neidhardt, Esther Glaus, Birgit Lorenz, Christian Netzer, Yün Li, Maria Schambeck, Mariana Wittmer, Silke Feil, Renate Kirschner-Schwabe, Thomas Rosenberg, Frans P.M. Cremers, Arthur A.B. Bergen, Daniel Barthelmes, Husnia Baraki, Fabian Schmid, Gaby Tanner, Johannes Fleischhauer, Ulrike Orth, Christian Becker, Erika Wegscheider, Gudrun Nürnberg, Peter Nürnberg, Hanno Jörn Bolz, Andreas Gal, Wolfgang Berger

**Affiliations:** 1 Division of Medical Molecular Genetics and Gene Diagnostics, Institute of Medical Genetics, University of Zurich, Switzerland; 2 Department of Paediatric Ophthalmology and Ophthalmogenetics, Universitätsklinikum Regensburg, Regensburg, Germany; 3 Department of Ophthalmology, Justus-Liebig-University Giessen, Universitaetsklinikum Giessen und Marburg GmbH Giessen Campus, Giessen, Germany; 4 Institute of Human Genetics, University of Cologne, Cologne, Germany; 5 Institute of Human Genetics, University Medical Center Hamburg-Eppendorf, Hamburg, Germany; 6 Charité – Universitätsmedizin Berlin, Department of Pediatric Oncology/Hematology, Berlin, Germany; 7 Gordon Norrie Centre for Genetic Eye Diseases, National Eye Clinic for the Visually Impaired, Hellerup, Denmark; 8 Department of Human Genetics, Radboud University Nijmegen Medical Centre, Nijmegen, The Netherlands; 9 Department of Ophthalmogenetics, The Netherlands Institute for Neuroscience, an institute of The Royal Academy of Art and Sciences (KNAW), Amsterdam, The Netherlands; 10 Department of Clinical Genetics, Academic Medical Centre (AMC), Amsterdam, The Netherlands; 11 Department of Ophthalmology, University Hospital Bern, Bern, Switzerland; 12 Department of Ophthalmology, University Hospital Göttingen, Göttingen, Germany; 13 Cologne Center for Genomics, University of Cologne, Cologne, Germany; 14 RZPD Deutsches Ressourcenzentrum für Genomforschung GmbH, Berlin, Germany; 15 Institute for Genetics, University of Cologne, Cologne, Germany

## Abstract

**Purpose:**

The goal of this study was to identify mutations in X-chromosomal genes associated with retinitis pigmentosa (RP) in patients from Germany, The Netherlands, Denmark, and Switzerland.

**Methods:**

In addition to all coding exons of *RP2*, exons 1 through 15, 9a, ORF15, 15a and 15b of *RPGR* were screened for mutations. PCR products were amplified from genomic DNA extracted from blood samples and analyzed by direct sequencing. In one family with apparently dominant inheritance of RP, linkage analysis identified an interval on the X chromosome containing *RPGR*, and mutation screening revealed a pathogenic variant in this gene. Patients of this family were examined clinically and by X-inactivation studies.

**Results:**

This study included 141 RP families with possible X-chromosomal inheritance. In total, we identified 46 families with pathogenic sequence alterations in *RPGR* and *RP2*, of which 17 mutations have not been described previously. Two of the novel mutations represent the most 3’-terminal pathogenic sequence variants in *RPGR* and *RP2* reported to date. In exon ORF15 of *RPGR*, we found eight novel and 14 known mutations. All lead to a disruption of open reading frame. Of the families with suggested X-chromosomal inheritance, 35% showed mutations in ORF15. In addition, we found five novel mutations in other exons of *RPGR* and four in *RP2*. Deletions in ORF15 of *RPGR* were identified in three families in which female carriers showed variable manifestation of the phenotype. Furthermore, an ORF15 mutation was found in an RP patient who additionally carries a 6.4 kbp deletion downstream of the coding region of exon ORF15. We did not identify mutations in 39 sporadic male cases from Switzerland.

**Conclusions:**

*RPGR* mutations were confirmed to be the most frequent cause of RP in families with an X-chromosomal inheritance pattern. We propose a screening strategy to provide molecular diagnostics in these families.

## Introduction

Retinitis pigmentosa (RP) is clinically characterized by night blindness, concentric constriction of visual fields, pigment deposits predominantly in the midperiphery of the retina, and attenuation of retinal vessels. It affects one in 3000–4000 individuals and can be caused by mutations in more than 40 genes (Retnet; Retina International). The disease may be inherited as autosomal recessive, autosomal dominant, or X-linked (XL) traits. Approximately 50% of the cases are sporadic [1].

Most mutations lead to a similar phenotype, making genotype-phenotype correlations difficult. Nevertheless, mutations in XLRP genes are associated with a severe phenotype in terms of onset and progression of the disease. The two known RP-associated X-chromosomal genes are designated *RPGR* (OMIM 312610) and *RP2* (OMIM 312600) [2-4]. Additional loci on the X-chromosome have been mapped, but the respective genes have not been identified [5-7]. Indeed, most XLRP cases can be explained by mutations in *RPGR* and *RP2* accounting for up to 80% and 20% of disease alleles, respectively. Moreover, *RPGR* mutations underlie 10–20% of all familial RP cases, which is higher than most other single RP loci [8,9].

The *RPGR* gene is composed of 23 exons, including the alternatively spliced exons 9a, ORF15, 15a, and 15b [10-12]. Exon ORF15 is predominantly found in transcripts of the retina and brain [13], whereas mRNAs including exons 1–19 are widely expressed [12]. The N-terminal part of RPGR contains a domain homologous to the regulator of chromosome condensation 1 (RCC1), the guanine-nucleotide-exchange factor for the GTPase Ran that has been shown to be important for the association with binding partners. *RPGR* mutations are most frequently found in exon ORF15, followed by exons 1–15 [9-12,14,15]. No mutations have been identified in exons 16–19 so far. ORF15 contains a purine-rich domain of approximately 1050 base pairs, which is predicted to encode a repetitive glycine and glutamate region at the C-terminus of the protein. In human, out of frame deletions, duplications, or insertions are frequently found in ORF15, whereas nonsense mutations are rare and disease-relevant missense mutations have not been described. Two canine animal models of ORF15 frame-shift mutations have been published and closely resemble the human phenotype in terms of disease onset and progression [16,17].

RT–PCR studies have suggested that splicing of both, constitutive exons and the ORF15-internal repeat of *RPGR*, generates numerous transcript variants [11,18]. This indicates that splicing is an important regulator of *RPGR* function. Recently, we have shown that a novel isoform of *RPGR*, including exon 9a, is expressed predominantly in cones of the human retina [10], which may explain why mutations in *RPGR* can also lead to a predominant degeneration of cones starting in the central part of the retina [19-22]. These findings extend the phenotypic spectrum associated with *RPGR* mutations and suggest a specific role of *RPGR* isoforms in the survival of cones and rods of the human retina.

*RP2* is composed of five coding exons, which are translated to a widely expressed protein of 350 amino acids [4]. The function of RP2 is not completely understood, but it shows homology to cofactor C, a protein involved in the ultimate step of gamma-tubulin folding, and interacts with ADP ribosylation factor-like 3 [23]. Posttranslational acyl modifications at the N-terminus of RP2 mediate its targeting to the plasma membrane, and the disruption of the acylation site leads to RP [24]. The majority of pathogenic sequence alterations found in RP2 represent truncating mutations, whereas missense mutations often locate to the cofactor C-like domain of RP2.

The present study describes the results of mutational screenings in RP families with possible XL inheritance patterns. In addition to known sequence alterations, we found several novel mutations in *RPGR* and *RP2* and characterized an XLRP family with variable disease expression in female carriers. We identified a microdeletion breakpoint in addition to an ORF15 frameshift mutation in a single RP patient. A screening strategy for molecular diagnostic testing in patients or families with presumed X-chromosomal inheritance is discussed.

## Methods

### Patients

Informed consent was obtained from each patient and healthy control after explanation of the nature and possible consequences of the study. Blood samples were collected to perform routine molecular genetic testing for genes associated with RP or related retinal diseases. We collected a panel of 141 retinal degeneration patients with possible XL inheritance patterns. Among those were 39 patients without history of additional affected family members, six cases with assumed dominant transmission and males preferentially affected, and 90 cases with possible XL-inheritance showing at least two affected male family member and no male-to-male transmission. The latter group also included male sibships. Six simplex cases were clinically diagnosed with cone-rod dystrophy, whereas all others were diagnosed with RP. Clinical evaluations of patients included slit lamp examination, funduscopy, and Ganzfeld-electroretinography (ERG). Ganzfeld-ERG was performed according to standards of the International Society for Clinical Electrophysiology of Vision using a LKC-UTAS 3000 ERG device (LKC Technologies Inc., Gaithersburg, MD) or a Nicolet Spirit examination unit (Nicolet, Madison, WI) [25,26]. For scotopic ERG recordings, we employed a phase of dark-adaptation preceded by single dim white and bright white flashes for stimulation. Bright white flashes were used for single responses and 30 Hz flicker responses in photopic ERG measurements. Goldmann perimetry (Goldmann perimetry module on the Octopus 101 visual field testing device; Haag-Streit, Köniz, Switzerland), fundus photography, and best corrected visual acuity testing were often performed to complement the clinical characterization.

Furthermore, we collected blood samples from 15 individuals of an XLRP family, segregating the disease in three generations (index 25085). In this family, ocular examinations, as approved by the ethics committee of the Institute of Human Genetics of the University Hospital of Cologne, were done in II:3, III:7, and III:12 and included refraction, best corrected visual acuity, intraocular pressure, slit-lamp biomicroscopy, funduscopy, and color discrimination test. ERG, dark adaptation and visual field were also analyzed in these three patients.

### Linkage analysis for an XLRP locus with variable heterozygote manifestation

We analyzed DNA samples from 15 individuals of a family that segregates RP in three generations (index patient 25085). PCR fragments of polymorphic microsatellite markers were amplified using fluorescent-labeled oligonucleotides and analyzed on an ABI-377 DNA sequencer (Applied Biosystems, Darmstadt, Germany). Genotypes were determined by GeneScan software (Applied Biosystems). Data provided by the GDB Human Genome Database were used as reference for allele sizes. Segregation analysis was performed by genotyping locus-specific microsatellite markers for the following AD RP genes: *NRL* (*RP27*), *CRX, RP1, PIM1K (RP9), IMPDH1 (RP10), CA4 (RP17)*, and *FSCN2*. Markers were not informative for *PRPF31, RDS,* and *RHO*. Therefore, the entire coding regions of the corresponding genes were sequenced in the index patient (III:7). In *PRPF3* and *PRPF8*, mutations have only been described in exons 11 and 42, respectively. These exons were directly sequenced. Primer sequences and PCR conditions are available upon request. The RP31 locus had not yet been described when this study was performed and thus, was not analyzed [27].

To map the disease locus, we performed a genome-wide linkage analysis using the Affymetrix GeneChip Human Mapping 10K Array, version 2.0 (Affymetrix, Santa Clara, CA). Genotypes were called by the GeneChip DNA Analysis Software (GDAS v2.0; Affymetrix). Genders of samples were verified by counting heterozygous single nucleotide polymorphisms (SNPs) on the X chromosome. Relationship errors were evaluated with the help of the program Graphical Relationship Representation [28]. The program PedCheck was applied to detect Mendelian errors [29], and data for SNPs with such errors were removed from the data set. Non-Mendelian errors were identified by using the program Merlin [30] and unlikely genotypes for related samples were deleted. Nonparametric linkage analysis using all genotypes of a chromosome simultaneously was performed with Merlin. Parametric linkage analysis was performed by a modified version of the program Genehunter 2.1 [31,32] through stepwise use of a sliding window with sets of 100 SNPs and by the program Allegro [33] assuming autosomal dominant inheritance with reduced penetrance and a disease allele frequency of 0.0001. Haplotypes were reconstructed with Allegro or Merlin and presented graphically with HaploPainter [34]. All data handling was performed using the graphical interface Alohomora [35].

### Mutation screening

The genomic DNA of the index patient panel was screened for disease-associated sequence alterations in exon ORF15 of *RPGR* by direct sequencing of PCR products. A single PCR fragment, spanning the entire coding region of exon ORF15, was amplified from 100 ng genomic DNA using primers ORF15-F3 and ORF15-R6 (Appendix 1). As described elsewhere, patients’ genomic DNA was either extracted by a salting out method or by affinity purification mediated by magnetic beads [36,37]. PCR was performed with either HotfireTaq (Solis Biodyne, Tartu, Estonia) or HotstarTaq Polymerase (Qiagen AG, Hombrechtikon, Switzerland) as recommended by the manufacturers. Q-solution and 3µM MgCl_2_ were added to the reaction mixture. Cycling conditions of the PCR were conducted for 45 cycles as follows: initial denaturation 95 °C for 15 min, denaturation 95 °C for 45 s, annealing 60 °C for 1 min, elongation 72 °C for 3.5 min. Subsequently, the PCR fragments were sequenced (ABI 3100; Applied Biosystems, Rotkreuz, Switzerland) with primers ORF15-R7b, -R8b, -R9, -R5, -R4, and -F10 (Appendix 1). The sequenced PCR profiles were compared to the *RPGR* reference database entry NM_001034853 (NCBI database). If genomic DNA were available only from female members of the family, sequence analysis of ORF15 was occasionally not complete, since polymorphic heterozygote deletions or duplications may resulted in overlapping sequence spectrums. If no mutations in *RPGR* exon ORF15 were detected, we subsequently analyzed all coding exons of *RP2* and the *RPGR* exons 1 through 15, 9a, 15a, and 15b, including flanking intronic sequences. *RP2* mutational screening was performed using either single strand conformation polymorphism analysis or direct sequencing [4] (Appendix 1). Long-range PCR was conducted as recommended by the manufacturers (Expand long range PCR system; Roche, Mannheim, Germany) using the primer ORF15-F10 and ORF15–15a-R10 (annealing temperature of 60 °C; elongation time of 10 min). Potential mutations of *RP2* or *RPGR* were verified by sequence analysis of the respective position in either 100 or 300 control alleles. The database entry BC043348 was used as reference sequence for *RP2* mutation analysis. The identified nucleotide substitutions and protein consequences are described as recommended (for guidelines see the Human Genome Variation Society) and, to be comparable to previously published data, as suggested by Sharon et al. [15], or Bader et al. [9].

### X-chromosome inactivation studies

A nonrandom X-inactivation may explain variable phenotypic expression in females that carry a single *RPGR* mutation. To test this possibility, we performed X-chromosome inactivation studies and examined the methylation patterns at the androgen receptor (AR) locus as described before [38]. Briefly, for each proband, we prepared two reactions. In the first, 400 ng genomic DNA were digested with HpaII (8 U) in a total volume of 10 μl for 30 min at 37 °C. In the second reaction, 150 ng genomic DNA were incubated without enzyme. Subsequent PCR amplification was done using oligonucleotide primers AR-A (5′-CTT TCC AGA ATC TGT TCC AG-3′) and AR-B (5′-AAG GTT GCC TGT TCC TCA TC-3′) in a PTC-thermal cycler (MJ Research, BioRad, München, Germany) at 95 °C for 1 min, 55 °C for 1 min, and 72 °C for 1 min for 35 cycles. About 2–10 μl of the product was mixed with 2–6 μl loading buffer and run at 70 W for 3–3.5 h on an 8% nondenaturing polyacrylamide gel. After electrophoresis, the gel was silverstained for evaluation. DNA samples of a male control as well as two female controls with previously confirmed unilateral X-inactivation were included into the analysis.

## Results

### Identification of mutations

**Figure 1:**
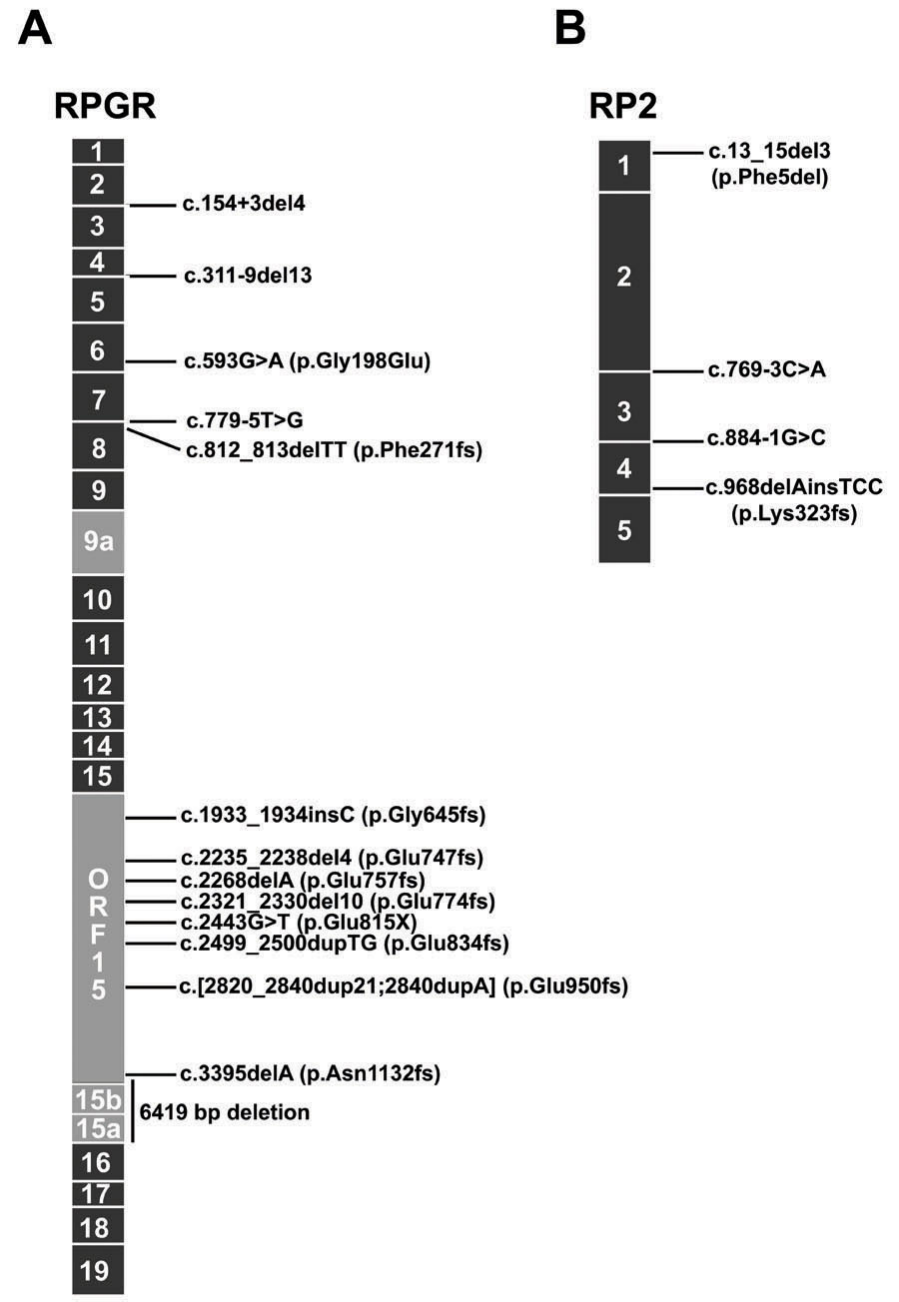
Location of novel *RPGR* and *RP2* mutations identified in this study. The schematic drawing shows novel mutations and their approximate positions in *RPGR* (**A**) and *RP2* (**B**). Alternatively spliced exons are shown in light gray, whereas other exons are depicted in black.

We analyzed DNAs from 141 patients with retinal degeneration for mutations in X-chromosomal RP-associated genes. DNA was collected from various geographical regions, including Germany, Switzerland, Denmark, and The Netherlands and contained 90 samples from families indicative for an XL-inheritance pattern. Within the subgroup of 90 index patients, we identified 31 (35%) mutations in exon ORF15 of *RPGR*. Among these ORF15 mutations, 14 sequence alterations had been reported previously, whereas eight were novel mutations (Figure 1A, Table 1). In addition to two nonsense mutations leading to a premature stop codon, we identified three different duplications, 16 distinct deletions, and one insertion. All cause a frameshift and were predicted to result in altered amino acids followed by a premature stop codon (Table 1). We excluded that additional nucleotide variations in *RP2* and *RPGR* may explain the phenotype of RP in patients with ORF15 mutations and performed mutation screenings in exons 1 through 15, 9a, 15a, and 15b of *RPGR* and all coding exons of *RP2*. In addition to pathogenic mutations, we identified several polymorphic sequence alterations (Table 2 and Table 3).

**Table 1:** Mutations found in *RPGR* and *RP2* in X-linked retinitis pigmentosa families

Patient ID	**Gene (exon/intron)**	**ORF15 mutation (1)**	**ORF15 protein change (1)**	**Mutation (2)**	**Protein change (2)**	**Mutation first reported by**
XRP51	RP2 (exon 1)	-	-	c.13_15del3	p.Phe5del	novel mutation
XRP6	RP2 (intron 2)	-	-	c.769-3C>A	-	novel mutation
XRP21*****	RP2 (exon 2)	-	-	c.358C>T	p.Arg120X	Hardcastle et al., 1999 [51]
XRP26	RP2 (intron 3)	-	-	c.884-1G>C	-	novel mutation
24748	RP2 (exon 4)	-	-	c.968delAinsTCC	p.Lys323fs	novel mutation
XRP11	RPGR (intron 2)	-	-	c.154+3del4	-	novel mutation
XRP19	RPGR (intron 4)	-	-	c.311-9del13	-	novel mutation
24750	RPGR (exon 6)	-	-	c.593G>A	p.Gly198Glu	novel mutation
XRP16	RPGR (exon 7)	-	-	c.644G>T	p.Gly215Val	Vervoort et al., 2002 [45]
24739	RPGR (intron 7)	-	-	c.779-5T>G	-	novel mutation
24746	RPGR (exon 8)	-	-	c.812_813delTT	p.Phe271fs	novel mutation
XRP32	RPGR (ORF15)	g.ORF15+180_181insC	ORF15Gly60fs	c.1933_1934insC	p.Gly645fs	novel mutation
XRP70	RPGR (ORF15)	g.ORF15+482_485delAGAA	ORF15Glu162fs	c.2235_2238delAGAA	p.Glu747fs	novel mutation
2460*****	RPGR (ORF15)	g.ORF15+483_484delGA	ORF15Glu161fs	c.2236_2237delGA	p.Glu746fs	Vervoort et al., 2000 [11]
24520	RPGR (ORF15)	g.ORF15+515delA	ORF15Glu172fs	c.2268delA	p.Glu757fs	novel mutation
2554	RPGR (ORF15)	g.ORF15+517_518delAG	ORF15Glu172fs	c.2270_2271delAG	p.Glu757fs	Sharon et al., 2003 [15]
XRP72	RPGR (ORF15)	g.ORF15+568_577delAGAGGAAAAA	ORF15Glu189fs	c.2321_2330delAGAGGAAAAA	p.Glu774fs	novel mutation
XRP48	RPGR (ORF15)	g.ORF15+587_588delAG	ORF15Arg195fs	c.2340_2341delAG	p.Arg780fs	Bader et al., 2003 [9]
25085° ****	RPGR (ORF15)	g.ORF15+652_653delAG	ORF15Glu217fs	c.2405_2406delAG	p.Glu802fs	Vervoort et al., 2000 [11]
XRP13	RPGR (ORF15)	g.ORF15+673_674delAG	ORF15Glu224fs	c.2426_2427delAG	p.Glu809fs	Vervoort et al., 2000 [11]
24751	RPGR (ORF15)	g.ORF15+690G>T(stop)	ORF15Glu230Ter	c.2443G>T	p.Glu815X	novel mutation
XRP27	RPGR (ORF15)	g.ORF15+714A>T (stop)	ORF15Lys238Ter	c.2467A>T	p.Lys823fs	Pelletier et al., 2006 [14]
2249	RPGR (ORF15)	g.ORF15+747_748insTG	ORF15Glu249fs	c.2499_2500dupTG	p.Glu834fs	novel mutation
24745	RPGR (ORF15)	g.ORF15+748delA	ORF15Glu249fs	c.2501delA	p.Glu834fs	Bader et al., 2003 [9]
2604	RPGR (ORF15)	g.ORF15+753delG	ORF15Glu251fs	c.2506delG	p.Glu836fs	Pelletier et al., 2006 [14]
10005°	RPGR (ORF15)	g.ORF15+795delG	ORF15Glu265fs	c.2548delG	p.Glu850fs	Prokisch et al., 2007 [52]
24731°	RPGR (ORF15)	g.ORF15+795delG	ORF15Glu265fs	c.2548delG	p.Glu850fs	Prokisch et al., 2007 [52]
2678	RPGR (ORF15)	g.ORF15+801_802insG	ORF15Glu267fs	c.2554dupG	p.Glu852fs	Bader et al., 2003 [9]
25333	RPGR (ORF15)	g.ORF15+833_834delGG	ORF15Glu278fs	c.2586_2587delGG	p.Glu863fs	Yokoyama et al., 2001 [53]
2865	RPGR (ORF15)	g.ORF15+875_876delGG	ORF15Glu292fs	c.2628_2629delGG	p.Glu877fs	Push et al., 2002 [54]
2814	RPGR (ORF15)	g.ORF15+1087_1088ins22	ORF15Glu365fs	c.[2820_2840dup21; 2840dupA]	p.Glu950fs	novel mutation
XRP23	RPGR (ORF15)	g.ORF15+1254_1257delGGAG	ORF15Gly418fs	c.3007_3010delGGAG	p.Gly1003fs	Sharon et al., 2003 [15]
24747**	RPGR (ORF15)	g.ORF15+1339delA	ORF15Glu446fs	c.3092delA	p.Glu1031fs	Bader et al., 2003 [9]
2557°°	RPGR (ORF15)	g.ORF15+1642delA	ORF15Asn547fs	c.3395delA	p.Asn1132fs	novel mutation

The table shows families with at least two affected males and no male to male transmission. A degree symbol (°) denotes cases with assumed dominant inheritance pattern. Two degree symbols (°°) show that in this patient an additional deletion was identified previously (Roepman et al., 1996) [39]. As shown in this study, the additional deletion does not remove parts of the coding region of exon ORF15 and deletes 6419 bp including exon 15a and 15b. Two asterisks (**) indicate that the mutation of this patient ID was identified two times. Four asterisks (****) and five asterisks (*****) show that the mutation was identified four and five times, respectively. The mutations are either presented in approved nomenclature according to Human Genome Variation Society (2) or as recommended by Bader et al., 2003 [9] and Sharon et al., 2003 [15] (1) (BC043348 and NM_001034853 were used as reference sequence for, respectively, *RP2* and *RPGR* mutation analysis).

**Table 2:** Polymorphic sequence variations in exon ORF15 of *RPGR* found in X-linked retinitis pigmentosa patients

**Patient ID**	**Deletions or duplications found in exon ORF15 (1)**	**Deletions or duplications found in exon ORF15 (2)**	**Nucleotide substitutions found in exon ORF15 (1)**	**Nucleotide substitutions found in exon ORF15 (2)**	**Protein change resulting from nucleotide substitutions (1)/(2)**
24731	g.ORF15+1060_1080dup21	c.2813_2833dup21	g.ORF15+1643C>T	c.3396C>T	p.N547N / p.N1132N
24731	-	-	g.ORF15+1677G>A	c.3430G>A	p.V559I / p.V1144I
24747	-	-	g.ORF15+1643C>T	c.3396C>T	p.N547N / p.N1132N
24747	-	-	g.ORF15+1677G>A	c.3430G>A	p.V559I / p.V1144I
2678	-	-	g.ORF15+1813A>C	c.3566A>C	3'UTR
2814	-	-	g.ORF15+1643C>T	c.3396C>T	p.N547N / p.N1132N
2814	-	-	g.ORF15+1677G>A	c.3430G>A	p.V559I / p.V1144I
2814	-	-	g.ORF15+1813A>C	c.3566A>C	3'UTR
2865	g.ORF15+1307_1318delAGTGGAAGGGGA	c.3060_3071del12	-	-	-
3044	g.ORF15+914_916delGGA	c.2667_2669del3	g.ORF15+588G>A	c.2341G>A	p.A196T / p.A781T
25085	g.ORF15+1307_1318delAGTGGAAGGGGA	c.3060_3071del12	-	-	-

The sequence alterations are either presented in approved nomenclature according to Human Genome Variation Society (2) or as recommended by Bader et al., 2003 [9] and Sharon et al., 2003 [15] (1) (BC043348 and NM_001034853 were used as reference sequence for, respectively, *RP2* and *RPGR* mutation analysis).

**Table 3:** Polymorphic sequence variations in exons 1 to 15 of *RPGR* found in X-linked retinitis pigmentosa patients

**Patient ID**	**Position in RPGR**	**Nucleotide substitutions found in RPGR exons 1 to 15**	**SNP ID**	**Heterozygosity frequency**	**Protein change**
24731	intron 1	c.29-15G>A	rs6651585	0.474 +/- 0.110	-
24745	exon 10	c.1164G>A	rs1801686	0.189 +/- 0.242	p.A388A
24747	intron 1	c.29-15G>A	rs6651585	0.474 +/- 0.110	-
24747	exon 9	c.1033A>G	-	0.007**	p.N345D
2249	exon 11	c.1367A>G	-	-	p.Q456R
2604	exon 10	c.1164G>A	rs1801686	0.189 +/- 0.242	p.A388A
2814	exon 9	c.1033A>G	-	0.007**	p.N345D
2814	intron 12	c.1507-101A>T	rs5918520	-	-
2557	intron 12	c.1507-101A>T	rs5918520	-	-
24520	intron 12	c.1507-101A>T	rs5918520	-	-
25428	intron 1	c.29-15G>A	rs6651585	0.474 +/- 0.110	-
25428	exon 9	c.1033A>G	-	0.007**	p.N345D
25428	intron 12	c.1507-101A>T	rs5918520	-	-
24748*	intron 4	c.310+10T>C	-	-	-

The sequence alterations are presented in approved nomenclature according to Human Genome Variation Society (NM_001034853 was used as reference sequence for mutation analysis). The asterisk (*) indicates that this sequence variant was not detected in over 300 control alleles. The two asterisks (**) indicate that this value was published by Sharon et al., 2000 [15].

The deletion c.2548delG in exon ORF15 of *RPGR* was detected in two independent families. In one of them (index patient 24731), the mother as well as three of her sons were affected by RP, whereas one son and one daughter were unaffected. Since the mother was affected, this suggests that female carriers of this mutation can be affected by RP. The second family, in which we found the mutation c.2548delG in the index patient 10005, showed variable disease manifestation in female carriers. The finding that two apparently unrelated families with the same *RPGR* mutation showed disease expression in female carriers, suggests a genotype-phenotype correlation. Further families with this mutation need to be analyzed to verify this observation.

**Figure 2:**
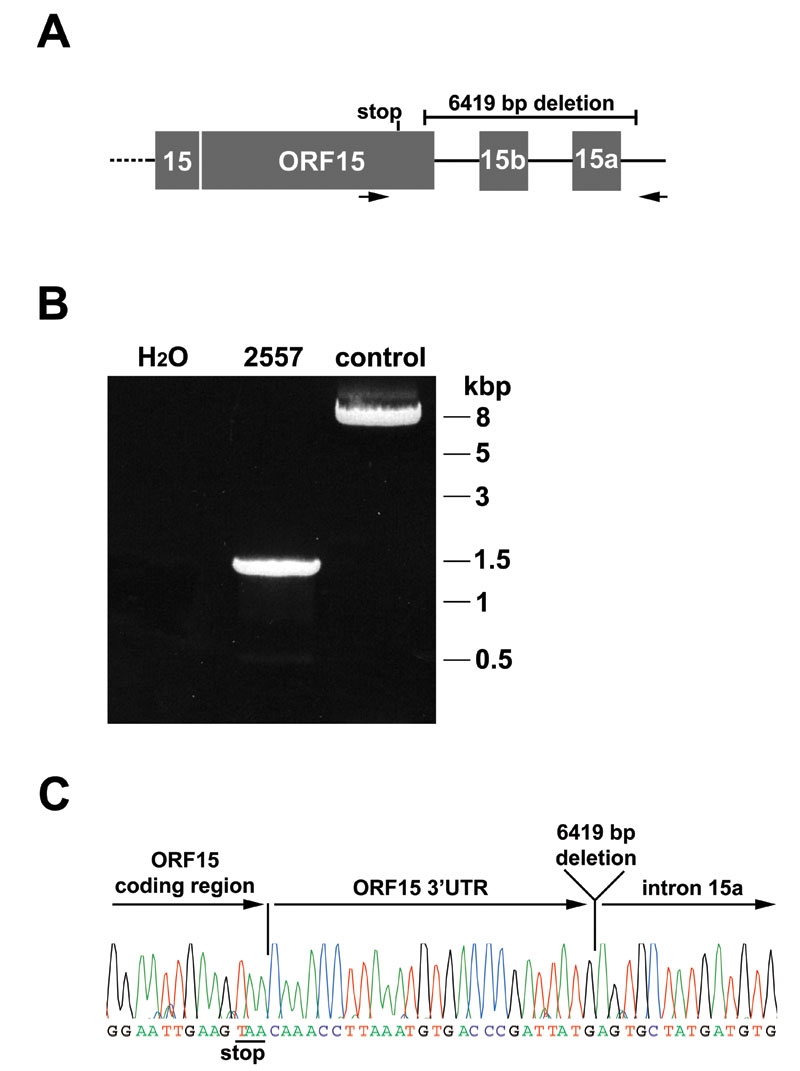
Characterization of the deletion breakpoint in patient 2557. **A:** Schematic drawing of *RPGR* exons ORF15, 15a, and 15b. Primers used for long-range PCR are marked by arrows and flank the 6419 bp deletion. **B:** Long-range PCR for patient 2557 (male) and a control sample (male). The difference of the PCR products represents the 6.4 kb deletion in patient 2557. **C:** The sequence profile shows the breakpoint of the deletion in patient 2557. The open reading frame of exon ORF15 was not affected by the deletion. In total, 6419 bp were deleted, starting 27 bp after the stop codon in ORF15 and ending 72 bp after exon 15a. Note that exon 15a is downstream of 15b. Thus, the deletion affected both exons 15a and 15b of *RPGR*.

In patient 2557, we identified the single bp deletion c.3395delA at the 3’ end of exon ORF15 (Table 1). Remarkably, the same patient had been shown to have an approximately 6.4 kb deletion in the *RPGR* gene [39]. We now have determined the breakpoints in the genomic DNA to verify whether the 6.4 kb deletion, in addition to c.3395delA, affects the exon ORF15 coding region. We performed long-range PCR and sequencing and found that the 6.4 kb deletion started 27 bp after the stop codon of exon ORF15 and ended 72 bp downstream of exon 15a (Figure 2A,B,C). Thus, the larger deletion of patient 2557 includes 6419 bp and removes exon 15a and 15b of *RPGR*, but does not affect the coding region of exon ORF15. In contrast, the mutation c.3395delA removes an adenine 62 bp upstream of the ORF15 stop codon. Consequently, the mutation was predicted to result in a frameshift at the 3’-terminal end of ORF15, which affects only the last 21 amino acids and results in a premature stop codon after inclusion of 19 RPGR-unrelated amino acids. The two deletions found in the male index patient 2557 locate to one allele at a distance of only 90 bp within *RPGR*.

In XLRP patients without an ORF15 mutation, we screened the five exons of *RP2* by direct sequencing and identified five pathogenic sequence variations, which either lead to a frameshift, an altered amino acid composition, or a presumed splice defect of RP2 (Figure 1B, Table 1).

DNA from index patients who did not show mutations in ORF15 and *RP2* were analyzed for sequence variations in exons 1 through 15, 9a, 15a, and 15b of *RPGR*. We were able to identify pathogenic sequence alterations in six additional XLRP cases. One mutation, found in exon 8, deletes two bp of codon 271, introducing a frameshift to the transcript. Three mutations affect intronic sequences close to *RPGR* exons and presumably result in splice defects. Furthermore, two mutations alter a glycine in either exon 6 or 7 of *RPGR* (Figure 1A, Table 1). None of these sequence alterations were identified in 100 to 300 control alleles. Sequence alterations summarized in Table 3 likely represent polymorphisms.

*RPGR* ORF15 mutations were neither detected in 39 simplex male cases with RP, nor in six simplex male cases affected by cone-rod dominated degenerations.

### Analyses of a retinitis pigmentosa family with an assumed dominant inheritance pattern

**Figure 3:**
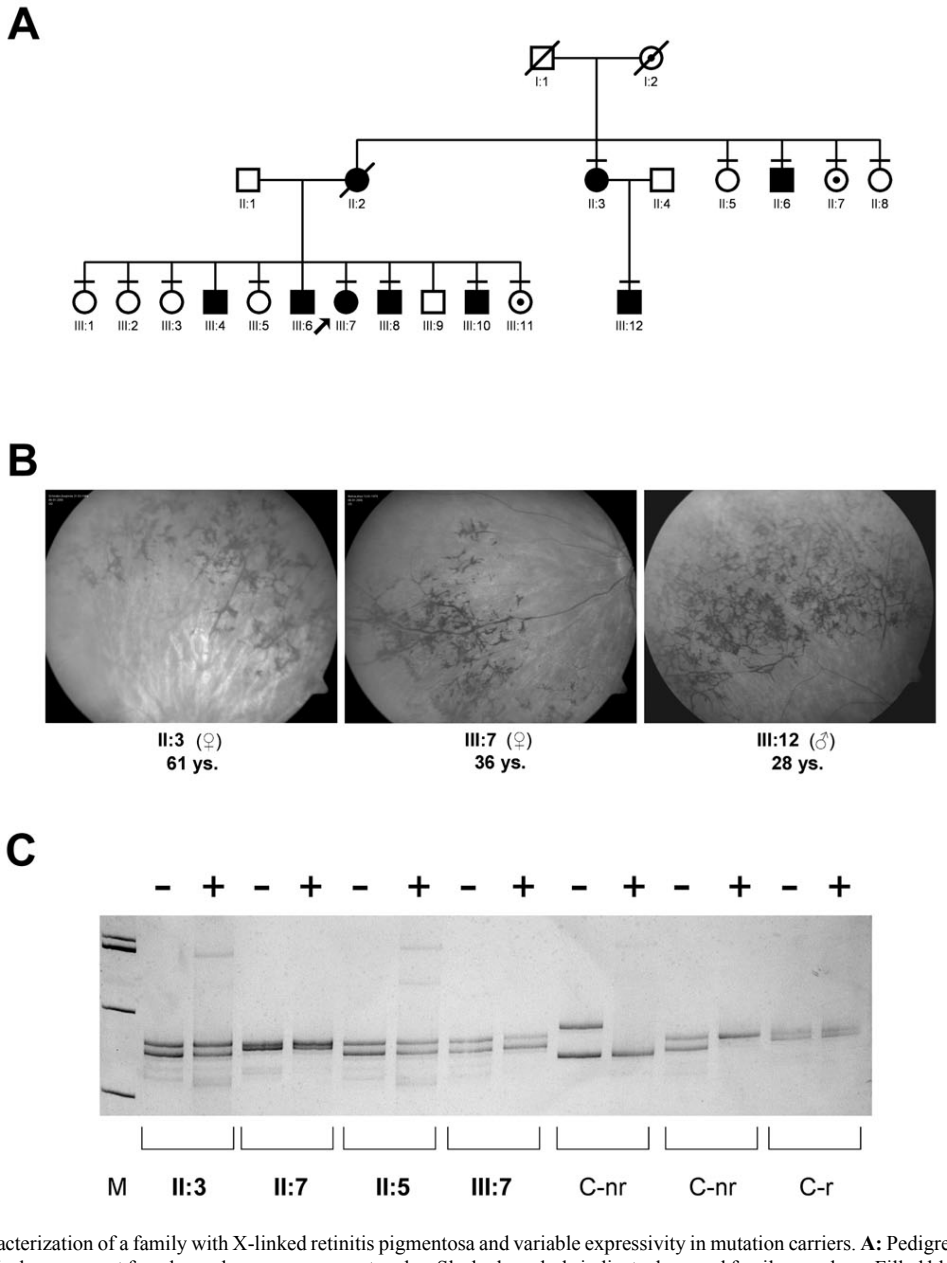
Characterization of a family with X-linked retinitis pigmentosa and variable expressivity in mutation carriers. **A:** Pedigree with three generations. Circles represent females and squares represent males. Slashed symbols indicate deceased family members. Filled black symbols denote family members with retinitis pigmentosa (RP), and circles with a dot indicate female mutation carriers who had no history of visual complaints. Horizontal bars designate family members whose genotype was determined by molecular genetic testing. Arrow marks the index patient 25085. The mutation c.2405_2406delAG in exon ORF15 of *RPGR* segregates with the disease in males, and shows variable heterozygote manifestation in females. **B:** Fundus pictures of three affected family members show typical pigmentations found in the peripheral retina of patients with RP. Patient age and gender are provided below each fundus photograph. **C:** Pattern of X-chromosome inactivation of selected female family members. None showed a unilateral X-inactivation at the AR-locus. The following abbreviations and symbols are used: control nonrandom X-inactivation (C-nr), control random X-inactivation (C-r), HpaII digestion (+), and no HpaII digestion (-).

Among the six families with dominant inheritance pattern, we found three deletions in exon ORF15 of *RPGR*. In one of these six families (index patient 25085, Figure 3A) linkage analysis by microsatellite genotyping was performed and excluded seven known ADRP loci. Sequencing of *PRPF31, RDS, RHO, HPRP3*, and *PRPF8* did not reveal causative mutations. Microarray-based linkage analysis suggested an 11.5 Mb region on the X-chromosome comprising the *RPGR* gene.

Subsequent analysis of *RPGR* identified a 2 bp deletion within the alternatively spliced exon ORF15 (c.2405_2406delAG, Table 1). This deletion was predicted to result in a frameshift, an altered amino acid composition, and a premature stop codon after 97 bp truncating the full length protein by 320 amino acids. In addition, an in-frame 12 bp deletion, previously described as a polymorphism [9], was found in each patient with the 2 bp deletion. This suggested that both deletions were located on the same allele. The 12 bp deletion was further downstream of the 2 bp deletion and affected positions c.3060_3071del12. No additional sequence alteration was identified in *RP2* and *RPGR*. The segregation of the deletions was in accordance with linkage data.

The mutation was identified in four males and four females of the family, whereas six healthy female relatives did not carry the mutation (Figure 3A). All male mutation carriers showed the RP phenotype, whereas only two of the four female carriers were affected (Figure 3A). In this family, the ORF15 mutation caused a variable heterozygote manifestation of the phenotype in females, although the majority of previously reported *RPGR* mutations are recessive.

Clinical evaluation and interviews with the patients showed that the course of the disease, with respect to age-of-onset and disease progression, varied both in affected males and females. Males reported awareness of visual impairment between seven and 24 years of age, and disease progression varied from slow to rapid. Females first noticed visual impairment at 24 years (II:2), 28 years (III:7), and 32 years (II:3). Individual II:3 (mother of III:12; 61 years old) rapidly lost vision within a few years, a disease course similar to the male family member III:4, who reported normal vision until age 24, followed by rapid visual loss. In contrast to the severe course of the disease in female II:3, kinetic perimetry, scotopic and photopic ERG, and dark adaptation indicated mild expression of RP in III:7 eight years after the diagnosis was made. However, she had pigmentary deposits in the midperiphery of her fundus (Figure 3B).

The phenotypic variability within female carriers of the family might be caused by an altered X-chromosomal inactivation. We analyzed the X-inactivation pattern in different family members, but did not find skewed signal intensities in any of the symptomatic female carriers (Figure 3C).

## Discussion

We identified 17 novel and 16 known mutations in *RPGR* and *RP2* by screening of 141 DNA samples from RP patients. In the subgroup of 90 possible XLRP families, including also male sibships, we identified five different *RP2* mutations and 28 distinct pathogenic sequence alterations in *RPGR*. Approximately 35% of the XLRP families described herein showed mutations in exon ORF15 of *RPGR*. This detection rate for ORF15 mutations was within the range found in other studies [8,14]. *RPGR* exon ORF15 mutations have been reported to occur either in 30% or 52% in North American families with XLRP [15,40], whereas 43% were reported for Spanish cohorts [41]. Furthermore, detection rates of 49% have been described for families from France and 60% for families from the UK/Ireland [11,14].

Of all published *RPGR* mutations, 55% have been reported to reside in exon ORF15 [1]. We detected a total of 37 *RPGR* mutations, including 31 located to exon ORF15. Thus, we identified over 80% of the *RPGR* mutations in ORF15, a proportion higher than described recently [1]. These variations in detection rates might be due to different ethnical backgrounds of the patient cohorts.

Up to 28% of sporadic XLRP cases from France were reported to carry mutations in exon ORF15 of *RPGR* [14]. In contrast, we did not find ORF15 mutations in 39 sporadic cases from Switzerland. This difference again indicates that the ethnical background of the cohorts influences the detection rate. Nevertheless, it might also be necessary to pre-select sporadic cases for early-onset RP phenotypes, as done by Pelletier et al. [14], to increase the chance to identify ORF15 mutations in sporadic cases.

In one XLRP family, we found the mutation c.968delAinsTCC in *RP2*. This pathogenic sequence alteration deletes a single nucleotide and inserts three novel nucleotides at the same position. It leads to a frameshift starting in exon 4 and was predicted to result in a premature stop in exon 5. Moreover, the mutation inserts three nucleotides in the penultimate position in exon 4 of *RP2* and thus alters the consensus sequence of the splice donor site. This might lead to mis-splicing with increased exon 4 skipping from *RP2* transcripts. The position of this mutation is, to the best of our knowledge, the most 3’-terminal disease-associated variant in RP2 identified so far. This also holds true for the mutation c.3395delA (patient 2557) in exon ORF15 of *RPGR*. In the same patient, an additional deletion of approximately 6.4 kb removing *RPGR* exons 15a and 15b was identified, a rearrangement that helped to identify the gene in 1996 [39]. In this study, we determined the breakpoint of this 6.4 kb microdeletion and found that the coding region of ORF15 was not affected. In contrast, the mutation c.3395delA (p.Asn1132fs) of patient 2557 leads to a frameshift in the 3’-terminal part of ORF15 and alters the composition of the last 21 amino acids of RPGR, which are not part of the repetitive and highly charged region in ORF15. Interestingly, 3’-terminal mutations in *RPGR* exon ORF15 are occasionally associated with cone-rod degenerations rather than classic RP [19-21,42]. However, in the case described herein, a typical RP phenotype was observed. It is not clear whether or not the microdeletion of over 6.4 kb influenced the phenotype in patient 2557.

Disease expression in males with *RPGR* mutations is severe. It has been shown previously that the phenotype of female carriers of pathogenic *RPGR* mutations is highly variable. Some exhibit severe symptoms, while others are asymptomatic. We have identified three pathogenic sequence alterations in the mutational hot spot exon ORF15 that lead to disease expression also in female carriers. The mutation c.2548delG was found in a family in which the mother, in addition to three of her sons, was affected. The same mutation occurred in a second family with reduced disease expression in female carriers (family 10005). The finding that a mutation leads to a phenotype only in a fraction of female carriers could be due to genetic modifiers. We identified additional in-frame variations in ORF15 that might influence the clinical picture in female carriers. Nevertheless, in-frame deletions and duplications were frequently described as polymorphisms of exon ORF15, which makes it unlikely to be the only factor causing the differences found in carriers. Other modifiers such as age, environmental factors, additional changes in the second allele of the carrier females, or genetic variability in other genes may influence the disease expression. Further investigations will identify and characterize the possible modifiers that determine the clinical outcome of *RPGR* mutations in female carriers. In contrast, no *RP2* mutations have been described that result into heterozygote carrier manifestations.

The mutation c.2405_2406delAG, found in one of the families with assumed dominant inheritance of this study, has been described previously [43,44]. The family characterized here showed severe retinal degeneration in males and mild (III:7) to severe (II:3) ocular symptoms in two female carriers. Moreover, we identified two female mutation carriers who had no history of visual complaints: II:7 (51 years old) and III:11 (29 years old). In accordance with Rozet et al. [43], we conclude that this mutation leads to variable heterozygote manifestation of the phenotype. With the assumption that the families are unrelated, this observation suggests a genotype-phenotype correlation associated with the *RPGR* mutation c.2405_2406delAG. We also identified an additional in-frame (12 bp) deletion in all carriers of the pathogenic 2 bp deletion. It is likely that the 12 bp deletion represents a polymorphism as described by Bader et al. [9], although an additional effect on the phenotype cannot be excluded. Skewed X chromosome inactivation as an explanation of the largely variable disease manifestations in females was not supported by our data. Full expression of disease symptoms in females with an *RPGR* mutation may be misleading in molecular analyses and genetics counseling. Thus, ORF15 mutations in *RPGR* should be excluded in families that seem to fit an AD pattern of RP, but lack male-to-male transmission.

We propose a screening strategy for routine molecular genetics testing of possible XLRP cases by direct sequencing. Between 30% and 80% of mutations are found in exon ORF15 of *RPGR* [40,45], followed by mutation frequencies that are similar for *RP2* and *RPGR* exons 1 through 15. In total, approximately 70%–80% of XLRP cases carry mutations in *RPGR* and up to 20% in *RP2*. Based on these results and to keep costs low, we recommend beginning with the screening of an XLRP male patient by ORF15 mutation analysis. Our approach to amplify the complete exon ORF15 in a single PCR reaction reduces costs and effort. After identification of an ORF15 mutation, the molecular diagnosis might be considered as confirmed, since patients with an additional mutation in exons 1 through 15 of *RPGR* and *RP2* have not been reported. Nevertheless, this cannot be excluded, and a complete screening of known XLRP genes should be offered. In addition, the segregation of the ORF15 mutation within the family might be verified. If no ORF15 mutation is identified, we recommend to continue the screen for mutations with *RP2* rather than *RPGR*. This recommendation is based on the fact that *RP2* is composed of five exons only, whereas 15 exons need to be screened to complete the sequence analysis of *RPGR* without any clear benefit in terms of likelihood to detect the causative mutation.

So far, no *RP2* mutation has been identified in families with likely dominant inheritance pattern, except for a single case, where the only affected female showed a structural chromosome aberration and a unilateral X-inactivation due to a balanced X-autosome translocation [14]. In contrast, dominant inheritance was reported for a couple of *RPGR* mutations [43,46]. Furthermore, ORF15 mutations have also been associated with phenotypes of the central part of the retina, including cone-rod or cone dystrophies and atrophic macular degeneration [19-21,42]. Consequently, in XLRP cases with manifestations of the phenotype in females or in patients with X-linked cone-dominated retinopathies, we recommend screening exon ORF15 first, followed by the other exons of *RPGR*.

In addition to the phenotype of RP, mutations in exons 6, 8, and 10 of *RPGR* have been associated with features of primary ciliary dyskinesia, hearing dysfunction, sinusitis, and recurrent infections [47-50]. Thus, in XLRP cases with additional phenotypes, the best candidate region of *RPGR* seems to be exons 1 through 15.

## Appendix 1: Primer combinations used to amplify and sequence *RPGR* or *RP2* exons including flanking intronic regions.

To access the data, click or select the words “Appendix 1”. This will initiate the download of a PDF file.
